# Predicting recovery after lumbar spinal stenosis surgery: A protocol for a historical cohort study using data from the Canadian Spine Outcomes Research Network (CSORN)

**DOI:** 10.1080/24740527.2020.1734918

**Published:** 2020-12-30

**Authors:** Erynne Rowe, Elizabeth Hassan, Lisa Carlesso, Janie Astephen Wilson, Douglas P. Gross, Charles Fisher, Hamilton Hall, Neil Manson, Ken Thomas, Greg McIntosh, Brian Drew, Raja Rampersaud, Luciana Macedo

**Affiliations:** a School of Biomedical Engineering, McMaster University, Hamilton, Ontario, Canada; b Department of Mechanical Engineering, McMaster University, Hamilton, Ontario, Canada; c School of Rehabilitation Science, McMaster University, Hamilton, Canada; d School of Rehabilitation, Université de Montréal, Montréal, Quebec, Canada; e Department of Surgery, McMaster University, Hamilton, Ontario, Canada; f Department of Physical Therapy, Faculty of Rehabilitation Medicine, University of Alberta, Edmonton, Alberta, Canada; g Vancouver General Hospital, University of British Columbia, Vancouver, British Columbia, Canada; h Department of Surgery, University of Toronto, Toronto, Ontario, Canada; i Canada East Spine Centre, Saint John, New Brunswick, Canada; j Cumming School of Medicine, University of Calgary, Calgary, Alberta, Canada; k Canadian Spine Society, Canadian Spine Outcomes Research Network, Toronto, Ontario, Canada

**Keywords:** lumbar spinal stenosis, back pain, longitudinal cohort study, surgery, partial least squares

## Abstract

**Background**: Symptomatic lumbar spinal stenosis (SLSS) is a condition in which narrowing of the spinal canal results in entrapment and compression of neurovascular structures. Decompressive surgery, with or without spinal fusion, is recommended for those with severe symptoms for whom conservative management has failed. However, significant persistent pain, functional limitations, and narcotic use can affect up to one third of patients postsurgery.

**Aims**: The aim of this study will be to identify predictors of outcomes 1-year post SLSS surgery with a focus on modifiable predictors.

**Methods**: The Canadian Spine Outcomes Research Network (CSORN) is a large database of prospectively collected data on pre- and postsurgical outcomes among surgical patients. We include participants with a primary diagnosis of SLSS undergoing their first spine surgery. Outcomes are measured at 12 months after surgery and include back and leg pain, disability (Oswestry Disability Index, ODI), walking capacity (ODI item 4), health-related quality of life, and an overall recovery composite outcome (clinically important changes in pain, disability, and quality of life). Predictors include demographics (education level, work status, marital status, age, sex, body mass index), physical activity level, smoking status, previous conservative treatments, medication intake, depression, patient expectations, and other comorbidities. A multivariate partial least squares model is used to identify predictors of outcomes.

**Conclusion**: Study results will inform targeted SLSS interventions, either for the selection of best candidates for surgery or the identification of targets for presurgical rehabilitation programs.

## RÉSUMÉ

**Contexte**: La sténose spinale lombaire symptomatique (SSL) est une affection dans laquelle le rétrécissement du canal rachidien entraîne le piégeage et la compression des structures neurovasculaires. La chirurgie décompressive, avec ou sans fusion vertébrale, est recommandée pour les personnes présentant des symptômes graves et pour lesquelles la prise en charge conservatrice a échoué. Cependant, une douleur persistante importante, des limitations fonctionnelles et l'usage de narcotiques peuvent affecter jusqu'à un tiers des patients après l'opération.

**Objectifs**: Cette étude aura pour but d’identifier les prédicteurs de résultats un an après l'intervention chirurgicale de la sténose spinale lombaire symptomatique, en mettant l'accent sur les prédicteurs modifiables.

**Méthodes**: Le Canadian Spine Outcomes Research Network (CSORN) est une vaste base de données de patients chirurgicaux collectées de manière prospective comprenant des données sur les résultats pré et postopératoires. Nous inclurons les participants ayant un diagnostic primaire de sténose spinale lombaire symptomatique, qui subissent leur première opération de la colonne vertébrale. Les résultats sont mesurés 12 mois après l'opération et comprennent les douleurs dorsales et aux jambes, le handicap (Oswestry Disability Index, ODI), la capacité de marche (ODI, point 4), la qualité de vie liée à la santé et un résultat composite de récupération globale (changements cliniquement importants dans la douleur, le handicap et la qualité de vie). Les prédicteurs comprendront des données démographiques (niveau d'éducation, situation professionnelle, état civil, âge, sexe, indice de masse corporelle), le niveau d'activité physique, le tabagisme, les traitements conservateurs antérieurs, la prise de médicaments, la dépression, les attentes des patients et d'autres comorbidités. Un modèle multivarié des moindres carrés partiels sera utilisé pour identifier les prédicteurs de résultats.

**Conclusion**: Les résultats de l'étude serviront de base à des interventions ciblées en matière de sténose spinale lombaire symptomatique, soit pour la sélection des meilleurs candidats à la chirurgie, soit pour déterminer des cibles pour les programmes de réadaptation pré-chirurgicale.

## Introduction

Symptomatic lumbar spinal stenosis (SLSS) is a condition with primarily a degenerative etiology in which narrowing of the spinal canal results in entrapment and compression of neurovascular structures.^1–3^ Patients with SLSS have leg pain, substantially diminished walking ability, back pain, high disability (high levels or pain related disability), and poor health-related quality of life (HRQoL).^1,4^ It is estimated that there is a 2% prevalence of LSS in people between 40 and 49 years and 11% in those 70 to 79 years of age.^3^ With an aging population, SLSS is a growing problem with similar levels of disability and impact on HRQoL as seen in those undergoing joint replacement surgery.^5^

The majority of patients with SLSS receive conservative interventions such as physiotherapy, steroid injections, and opioids.^2^ Decompressive surgery is recommended for those with intolerable SLSS-related pain and/or functional limitations for whom conservative management has failed. Instrumented spinal fusion is usually reserved for patients with SLSS with associated deformity or instability, and these procedures have significant risk of complications.^6^ Unfortunately, significant persistent pain, functional limitations, diminished HRQoL, and narcotic use can affect up to one third of patients postsurgery.^7–9^ More specifically, there is evidence to suggest that approximately 30% of patients do not reach a minimal clinical important change in disability, pain, or quality of life 1 year postsurgery.^10–12^ Further, a recently published large population-based study identified that more than 40% of patients undergoing fusion for SLSS remain long-term opioid users.^13,14^

A number of studies have evaluated predictors of postsurgical outcomes, including a systematic review published in 2006.^15^ Poor surgical outcomes as related to disability, pain, walking capacity, or HRQoL may include an array of potential predictors, such as frailty, obesity, smoking, recovery expectations, depression, opioid use, better walking capacity, and lower pain at baseline as well as higher education level and socioeconomic status, age, sex, and comorbidities.^11,15–18^ A limitation of these studies is that they are unable to account for a large number of predictors because of the likelihood of multicollinearity and therefore generally only include a limited number of factors in their models.^11,12,15–18^ In addition, as with many studies in SLSS, outcomes used in prediction analysis are variable, which means that it is difficult to make generalized conclusions. A major advantage of the current protocol is that it allows for a stable and simultaneous analysis of multiple outcomes with a large number of predictors using core back pain outcomes.^19^

As previously mentioned, the literature could be improved upon by utilizing sophisticated statistical modeling on very large, high-quality data sets to identify modifiable predictors. Personalized management strategies to identify best candidates for surgery and the development of a presurgical rehabilitation program may improve patient outcomes. Thus, our primary aim is to identify predictors of back and leg pain, disability, walking capacity, HRQoL, and clinically important change across all outcomes (recovery) 1-year post SLSS surgery.

## Methods

### Study Design

This is a historical cohort study using data from the Canadian Spine Outcomes Research Network (CSORN) registry.^20–22^ The STROBE (Strengthening the Reporting of Observational Studies in Epidemiology) checklist is used for reporting of the study and was used to construct the protocol.^23^ This study received ethics approval from the Hamilton Integrated Research Ethics Board (HiREB #7285-C). The CSORN is a large database consisting of spine surgical data collected from patients of more than 50 neurosurgeons and orthopedic spine surgeons at 18 sites across Canada. It includes data pre- and postsurgery that were collected from patients diagnosed with a variety of different spinal pathologies, including SLSS. Data collection is conducted at baseline (pre-op), as well as at 3 and 12 months postoperatively. Standardized questionnaires are used to collect information on demographics and comorbidities and include lifestyle questions such as physical activity level and work status, past and current management strategies as related to the condition, self-reported expectations of outcomes, and pain, disability, and HRQoL as outcome measures. Surgeons also record surgical information such as specific procedure, complications, and length of hospital stay. All 18 sites that contributed to the registry obtained research ethics board approval prior to any data collection. Recently, the CSORN steering committee implemented improvements to their data collection procedures to improve data completeness; thus, only data from January 2015 to September 2019 will be utilized in this study.

### Participants

The inclusion criteria for this study include two factors:

1. Patients must have a primary diagnosis of SLSS provided by the treating spinal surgeon. Diagnosis was provided based on the surgeon’s assessment and clinical judgment because there are no clearly established diagnostic criteria.
2. Availability of 12-month postoperative CSORN outcome data (pain, disability, and quality of life).

Exclusion criteria include previous history of spinal surgery (self-reported), as well as low levels of back and leg pain (less than 3 on a 0–10 scale), low levels of disability (<20% on the Oswestry Disability Index [ODI]), and high quality of life (<20% on the Health Utility Index) measured at baseline. There were no exclusions in relation to the length of symptoms or comorbidities. These are included in our analysis as potential predictors. We did not exclude participants based on the presence of additional imaging findings such as disc herniation or degenerative disc disease.

### Outcomes

Six different outcome measures are included in this study, including back pain, leg pain, disability, walking capacity, HRQoL, and clinically significant change. Back and leg pain were measured using a numeric rating scale (NRS). The NRS is one of the most frequently used instruments to measure low back pain and is currently a core outcome measure in the last low back pain outcome measures consensus.^19^ The NRS is a scale ranging from 0 (*no pain*) to 10 (*worst possible pain*), with patients indicating their current pain intensity. Disability was measured using the ODI, a condition-specific outcome measure for spine- and back-related disorders that presents a subjective percentage score of a patient’s level of function (scored from 0 to 100).^24^ The ODI is also a core outcome measure in low back pain with significant evidence for validity, reliability, and responsiveness.^17,19^ Walking capacity is assessed using item 4 of the ODI. This self-reported question assesses a patient’s ability to walk various distances (pain does not prevent me walking any distance, pain prevents me walking more than 100 meters, pain prevents me walking more than 500 meters, pain prevents me walking more than 1 kilometer, I can only walk using a stick or crutches, I am in bed most of the time and have to crawl to the toilet). This question is often used in SLSS literature, has been found to have good evidence for responsiveness,^25^ and is a recommended outcome from a recent systematic review of walking tests in SLSS.^26^ HRQoL was measured using the EQ-5D-5L, which is a questionnaire describing the patient’s health state using an index value system (scored from 0 to 100, with 100 indicating perfect health).^27^ This instrument utilizes a value set that weighs each health state description according to the preferences of the general population of a region.^28^ Overall recovery composite outcome is used as a surrogate measure of recovery and is assessed using a combination of clinically important change in pain, disability, and HRQoL.^29^ A patient is deemed fully recovered if he or she meets all four criteria outlined in Table 1. This was used because it has been recommended for low back pain and no similar index has been indicated for surgical populations. The cutoffs for mild levels of pain, disability, and HRQoL used to define recovery are summarized in Table 1.^30^

**Table 1.** Cutoff scores of each outcome variable used to define the recovery composite score

Outcome	Cutoffs used to define recovery
Leg pain	Mild pain (0–3) on an NRS scale at 12 months
Back pain	Mild pain (0–3) on an NRS scale at 12 months
Disability	<30% on the ODI
Quality of life	<30% on the EQ-5D-5L index value

NRS = numeric rating scale; ODI = Oswestry Disability Index.

### Potential Predictors

Predictors were chosen for the present study based on data available from the CSORN registry, previous literature, and clinical assumptions. Factors that have been identified to be associated with improved disability outcomes include higher education level, higher quality of life (EQ-5D) at baseline, lower disability (ODI) at baseline, shorter duration of back pain,^17^ and lower levels of obesity (body mass index < 30).^11^ Shorter duration of symptoms prior to surgery is also positively correlated with reduced pain postsurgery.^15^ Factors that have been identified in the literature as being associated with improved walking capacity outcomes include younger age, male sex, higher reported walking capacity at baseline, lower levels of back and leg pain at baseline,^16^ and better self-rated health at baseline.^15,16^ Factors that have been identified to be associated with poor postsurgical outcomes include depression (in this study measured using the Patient Health Questionnaire-9), high levels of back pain at baseline, higher expectations of pain relief expectations going into surgery,^15^ cardiovascular comorbidity,^12^ and smoking, living alone, and unemployment.^17^ The data available from the CSORN registry include many of these predictors. The predictors we have chosen to include in our models are listed in Table 2.

**Table 2.** Predictor variables and variable codes

Predictor variable	Code
Sex	Male, female
Age	(Continuous), years
Body mass index	(Continuous), kg/m^2^
Duration of complaint	<6 weeks, 6–12 weeks, 3–6 months, 6–12 months, 1–2 years, >2 years
Marital status	Married, not married
Education level	Less than high school, high school, technical, college, postgraduate
Smoking status at the time of surgery	Smoker, nonsmoker
Physical activity level (How often do you exercise?)	Never, ≤1× week, ≥ 2× per week
Current work status	No change in work status due to LSS, modified duties, short-term disability, long-term disability
Previous treatments in the last 6 months (chiropractor)	Never seen, 1–2 times, 3–30 times, >30 times
Previous treatments in the last 6 months (physiotherapy)	Never seen, 1–2 times, 3–30 times, >30 times
Previous treatments in the last 6 months (trainer)	Never seen, 1–2 times, 3–30 times, >30 times
Medication intake (narcotic, nonsteroidal anti-inflammatory, antidepressant, neuroleptic)	Never, intermittent, daily
Patients’ expectations or hope for changes in back pain, leg pain, independence with ADL, sports and recreation, physical capacity, social activities and mental well-being	Each of the seven categories rated from 0 to 4 (*I don’t know, no change, somewhat better, better*, and *much better*)
Depression (PHQ9)	(Continuous) total score on scale from 0 to 27
Number of comorbidities (e.g., cardiovascular, diabetes, cancer)	(Continuous) total score from 0 to 24

LSS = lumbar spinal stenosis; ADL = activities of daily living; PHQ9 = Patient Health Questionnaire-9.

### Statistical Analysis

The goal of this analysis is to identify the predictors that are most relevant to the six identified outcome measures. The outcomes (back pain, leg pain, disability, walking capacity, HRQoL, and clinical recovery) are likely correlated, and the large number of predictors would probably suffer from multicollinearity in a multiple regression model. Therefore, we use a multivariate approach, partial least squares (PLS),^31^ that allows for the simultaneous analysis of multiple outcomes with a large number of predictors and is stable even when the input data have moderate levels of collinearity. PLS is a technique that combines principal component analysis with multiple linear regression. The mix of categorical and continuous predictor variables requires the use of a modified version of the PLS algorithm, partial least squares correspondence analysis.^32^ This technique has been used for neuroimaging and genetics data sets. The set of predictors are listed in Table 2, and the set of dichotomous outcomes is provided in Table 1.

Prior to the analysis, the CSORN data were preprocessed using the table functions in MATLAB; for example, to compute total scores or identify rows with missing data. As part of our inclusion criteria, we excluded any patient who did not complete 12-month follow-up. When data were missing for predictors and covariates (at a maximum of 20%), we used multiple imputation and conducted a sensitivity analysis after removing rows with missing data. However, when data were missing for outcomes (e.g., a patient completed health-related outcomes but not disability at 12 months postsurgery), the patient record was excluded from the analysis of multiple outcomes.

An objective criterion was used to eliminate predictors from the model to achieve a more parsimonious model. An example of such a criterion is iterative variable importance for projection.^33^ This method identifies the least important variable, eliminates that variable, reruns the analysis, and repeats until the desired balance of parsimony and prediction is achieved.

The PLS model was cross-validated by a hold-out procedure. This step prevents overfitting the model to extreme observations. The data are split into two sets, a training set and a test set, with set membership being random. The PLS model building step was repeated with the training set, and ability of the model to predict test set observations was evaluated. This was repeated 1000 times to ensure that every combination of observations was tested.

Concomitantly to the PLS, we also conducted traditional regression analysis for each independent outcome. We used multiple linear regression for the continuous outcomes of pain, disability, and HRQoL; logistic regression for the outcome of recovery; and ordinal regression for the outcome of walking capacity (ODI item 4). Assumptions and multicollinearity were assessed as appropriate. Regression was conducted in Stata 14.0 with a significance level of 0.05. Following Sex and Gender Equity in Research guidelines, we conducted sex-specific analyzes (sex as per collected in the CSORN) by including sex as a cofounder in the total model and performing a disaggregated analysis by sex.^34^

We conducted an a priori sample size calculation considering the baseline risk for our primary outcome of disability (assessed using the ODI). Although this outcome is continuous, we dichotomized the outcome as per the overall recovery composite outcome. As per Peduzzi et al., we included at least 20 patients (10 events, 10 nonevents) per predictor category.^35^ With a total of 16 predictors, we needed to include at least 160 fully recovered and 160 unrecovered patients in the model. Thus, based on the literature that 30% of patients do not recover from pain-related disability following surgery,^12,15,18^ our logistic regression model required a minimum sample size of 540 participants. However, we included at least 10 patients per predictor category as is customary for creating robust multivariable models.

## Discussion

SLSS treatment outcomes, whether conservative or surgical, are variable, with a large number of patients continuing to have significant levels of pain, disability, and diminished HRQoL. To date, interventions are delivered based on health care professionals’ expertise without much guidance on what treatment may be best for different patient subgroups. Recognizing the impact that this disorder has on the lives of the patients, it is imperative to develop better treatment approaches, treatment pathways, and personalized care. Modifiable predictors such as smoking, physical activity level, medication intake, and patients’ expectations could all be addressed in a prehabilitation program and potentially lead to improved patient outcomes.

### Strengths

The strengths of this study are the large sample size and data collected within usual clinical practice that reflect the Canadian context. Additionally, the robust, multivariate statistical analysis using PLS allows for the inclusion of predictors that are collinear within the model and the identification of patients who fit and do not fit within the predicted outcomes. This analysis also allows for the inclusion of multiple outcomes within a single model, potentially identifying patient phenotypes and their predictors (e.g., high pain, low function). This modeling approach provides important insight into the complex relationships between predictors and outcomes that will allow for more person-specific prehabilitation by identifying subgroups of patients with similar pathways. Additionally, a comprehensive use of the Sex and Gender Equity in Research guidelines for reporting sex differences allows for a better understanding of the role of sex given previous conflicting evidence reported in a systematic review of predictors of outcomes.^15^

### Limitations

The CSORN database is a valuable resource with a large number of participants, allowing for the inclusion of a large number of predictors and outcomes. However, there is always inherited bias when using registry data, such as the potential for large numbers of missing data, leading to high attrition bias and potential selection bias. Recent support from the Canadian Spine Society has resulted in improved data collection within the CSORN registry. Thus, we decided to exclude data collected prior to January 2015 in order to increase the quality and completeness of available data and thus reduce the risk of bias. Additionally, we attempted to reduce the risk of potential bias by using multiple imputation and a sensitivity analysis for missing predictor data in order to reduce the amount of data eliminated from the analysis. In addition, data analysis was limited to data available within the database as well as the format and measures used to collect the outcomes. Nonetheless, the knowledge gained through the proposed approach will better inform prospective and treatment trials to further our understanding of modifiable factors that predict poor postsurgical outcomes for SLSS.

### Conclusion

This protocol describes the first study of a Canadian surgical database to identify modifiable factors associated with pain, disability, HRQoL, and recovery in patients with SLSS. It is anticipated that the knowledge gained from the study described within this protocol will facilitate clinical decision making for managing patients with SLSS and inform the development of a prehabilitation program.
